# The Association between COVID-19 Infection and Kidney Damage in a Regional University Hospital

**DOI:** 10.3390/medicina59050898

**Published:** 2023-05-08

**Authors:** Giedrė Žulpaitė, Laurynas Rimševičius, Ligita Jančorienė, Birutė Zablockienė, Marius Miglinas

**Affiliations:** 1 Faculty of Medicine, Vilnius University, M. K. Ciurlionio 21, 03101 Vilnius, Lithuania; 2 Clinic of Gastroenterology, Nephrourology and Surgery, Institute of Clinical Medicine, Faculty of Medicine, Vilnius University, M. K. Ciurlionio 21, 03101 Vilnius, Lithuania; laurynas.rimsevicius@santa.lt (L.R.); marius.miglinas@santa.lt (M.M.); 3 Clinic of Infectious Diseases and Dermatovenerology, Institute of Clinical Medicine, Faculty of Medicine, Vilnius University, M. K. Ciurlionio 21, 03101 Vilnius, Lithuania; ligita.jancoriene@santa.lt (L.J.); birute.zablockiene@santa.lt (B.Z.)

**Keywords:** acute kidney injury, chronic kidney disease, COVID-19, kidney replacement therapy

## Abstract

*Background and Objectives*: Kidneys are one of the main targets for SARS-CoV-2. Early recognition and precautionary management are essential in COVID-19 patients due to the multiple origins of acute kidney injury and the complexity of chronic kidney disease management. The aims of this research were to investigate the association between COVID-19 infection and renal injury in a regional hospital. *Materials and Methods*: The data of 601 patients from the Vilnius regional university hospital between 1 January 2020 and 31 March 2021 were collected for this cross-sectional study. Demographic data (gender, age), clinical outcomes (discharge, transfer to another hospital, death), length of stay, diagnoses (chronic kidney disease, acute kidney injury), and laboratory test data (creatinine, urea, C-reactive protein, potassium concentrations) were collected and analyzed statistically. *Results*: Patients discharged from the hospital were younger (63.18 ± 16.02) than those from the emergency room (75.35 ± 12.41, *p* < 0.001), transferred to another hospital (72.89 ± 12.06, *p* = 0.002), or who died (70.87 ± 12.83, *p* < 0.001). Subsequently, patients who died had lower creatinine levels on the first day than those who survived (185.00 vs. 311.17 µmol/L, *p* < 0.001), and their hospital stay was longer (Spearman’s correlation coefficient = −0.304, *p* < 0.001). Patients with chronic kidney disease had higher first-day creatinine concentration than patients with acute kidney injury (365.72 ± 311.93 vs. 137.58 ± 93.75, *p* < 0.001). Patients with acute kidney injury and chronic kidney disease complicated by acute kidney injury died 7.81 and 3.66 times (*p* < 0.001) more often than patients with chronic kidney disease alone. The mortality rate among patients with acute kidney injury was 7.79 (*p* < 0.001) times higher than among patients without these diseases. *Conclusions*: COVID-19 patients who developed acute kidney injury and whose chronic kidney disease was complicated by acute kidney injury had a longer hospital stay and were more likely to die.

## 1. Introduction

On 11 March 2020, the World Health Organization declared the SARS-CoV-2 outbreak a global pandemic [1]. More than 670,000,000 cases of COVID-19 infection and more than 6,700,000 deaths caused by this infection have already been reported worldwide. In Lithuania, more than 1,200,000 cases and 9500 deaths have occurred [2]. COVID-19 has emerged as one of the most daunting diseases of the 21st century, presenting a grave danger to the health and wellbeing of humanity [3].

The COVID-19 virus is transmitted by airborne droplets [4]. The clinical spectrum of SARS-CoV-2 infection is extensive: it includes the asymptomatic carriage of the virus, mild upper respiratory tract disease to severe acute respiratory distress syndrome (ARDS), multiple organ failure, and death [5]. Acute kidney injury (AKI) is one of the most common complications of COVID-19 infection: proteinuria occurs in more than 40% of hospitalized patients [6], and according to European and US studies, AKI develops in approximately 50% of COVID-19 patients treated in the intensive care unit (ICU) [7], which is one-sixth of all hospitalized COVID-19 patients [8]. Approximately 20% of COVID-19 patients admitted to ICUs require renal replacement therapy [5]. According to a meta-analysis, the risk of death in patients who develop AKI is 13 times higher than in patients who do not develop AKI [9]. Recent data have also shown that chronic kidney disease (CKD) and renal replacement therapy are important risk factors for mortality in patients with COVID-19 [10]. Thus, kidney damage during COVID-19 infection is a major adverse factor in disease severity and survival [10]. Furthermore, kidney damage during COVID-19 infection leads to higher treatment costs [11,12]. The multiple origins of acute kidney injury and complexity of the management of chronic kidney disease in COVID-19 patients make early recognition and a more cautious approach essential. In this research we aimed to investigate the association between COVID-19 infection and renal injury in a regional hospital.

## 2. Materials and Methods

### 2.1. Data

We performed a cross-sectional study using the Vilnius regional hospital’s database. We obtained data for 601 patients treated between 1 January 2020 and 31 March 2021. Patients were included if they fulfilled the following criteria: at least 18 years of age, infected with SARS-CoV-2, confirmed by a molecular test, suffering from kidney disease (AKI, CKD, nephritic syndrome, nephrotic syndrome, glomerulonephritis, other changes due to impaired function of renal tubules, patients after kidney transplantation). We extracted demographic data (gender, age), clinical outcomes (hospital discharge to home, discharge from emergency department, transferred to another hospital for an active inpatient treatment or supportive care, death), duration of treatment, diagnoses (chronic kidney disease, acute kidney injury), and laboratory tests (creatinine, urea, C-reactive protein, potassium concentrations) from the electronic medical records. Biochemical parameters—creatinine, urea, C-reactive protein—were measured in the serum and potassium in the plasma samples using standard commercially available biochemical kits, following the manufacturer’s instructions. Analyses were performed on an automated analyzer in the hospital’s laboratory. All data used in this study were collected and analyzed in an anonymized manner, ensuring that the privacy and confidentiality of the participants were maintained throughout the research process.

### 2.2. Data Structure

For this cross-sectional study, we collected data based on relational data tables. Each patient was assigned a unique case identification number. The data were systematized, grouped, and coded using the Microsoft Excel 365 program.

### 2.3. Statistical Methods

Collected data were analyzed statistically using the R Commander software package. A Kolmogorov–Smirnov test was used to determine the distribution of quantitative variables, Spearman, Kendal, and Pearson tests were used for correlation between variables, Student’s *t*-test was used to compare the averages of equal variables, and the Mann–Whitney test was used for unequal ones. The chi-square test was used for the analysis of qualitative variables (for categorical variables) and ANOVA for multiple estimates for categorical variables. The difference between indicators was considered statistically significant when *p* < α (α = 0.05).

## 3. Results

### 3.1. Patients

Six hundred and one patients met the criteria and participated in the study. Table 1 displays the general characteristics of the study participants.

We found that the outcome was different according to the age of the COVID-19 patients (*p* < 0.001). Patients discharged from the hospital were younger (63.18 ± 16.02 years) than non-hospitalized emergency patients (75.35 ± 12.41 years, 95% CI (−18.40; −5.94), *p* < 0.001), those transferred to another hospital for further active inpatient treatment (72.89 ± 12.06 years, 95% CI (−16.98; −2.44) *p* = 0.002), those who died in hospital (70.87 ± 12.83 years, 95% CI (−11.37; −4.01), *p* < 0.001), or those transferred to a supportive care hospital (80.42 ± 7.08 years, 95% CI (7.56; 26.92) *p* < 0.001).

Patients whose treatment in the regional hospital ended in death had a shorter hospital stay (17.31 ± 20.38 days) than patients who were discharged (26.43 ± 25.42 days, 95% CI (3.32; 14.93), *p* < 0.001) or were transferred to a nursing hospital (33.21 ± 24.96 days, 95% CI (0.70; 31.11), *p* = 0.034). We found that the outcome of COVID-19 patients was independent of gender (*p* = 0.543).

### 3.2. Laboratory Findings

#### 3.2.1. Creatinine

Patients had their creatinine levels measured within the first day after admission to the regional hospital. We found that higher creatinine concentration correlated with older age (rh Spearman’s correlation coefficient = 0.133, *p* = 0.028) but shorter duration of treatment (Spearman’s correlation coefficient = −0.304, *p* < 0.001) (Table 2). Interestingly, the first-day creatinine level was higher in patients whose hospitalization outcome was death compared to discharged patients (185.00 ± 193.74 vs. 311.17 µmol/L, 95% CI (36.65; 215.70), *p* < 0.001). This can be explained by the difference in first-day creatinine levels in patients with chronic kidney disease and acute kidney injury (365.72 ± 311.93 vs. 137.58 ± 93.75 µmol/L, 95% CI (−303.37; −152.92), *p* < 0.001). No significant differences in creatinine levels were found between genders.

#### 3.2.2. Urea

Urea levels were measured within the first 24 h of the patients’ arrival at the regional hospital. Higher urea levels correlated with older age (Spearman’s correlation coefficient = 0.326, *p* < 0.001) and shorter duration of treatment (Spearman’s correlation coefficient = −0.247, *p* < 0.001) (Table 2). There was no significant difference in urea levels between patients with different outcomes or gender.

#### 3.2.3. Potassium

Potassium concentrations were also measured in the first 24 h, but no statistically significant differences or correlations were found (Table 2).

#### 3.2.4. C-Reactive Protein

CRP concentration during the first day did not correlate with age or duration of treatment (Table 2). However, CRP levels on the first day were higher in patients who were discharged home after hospitalization than in those who were transferred to another hospital (102.70 ± 96.66 vs. 161.41 ± 124.05 mg/L, 95% CI (−115.50; −1.91), *p* = 0.038) or died (102.70 ± 96.66 vs. 153.12 ± 109.59 mg/L, 95% CI (−78.37; −22.47), *p* <0.001).

### 3.3. Chronic Kidney Disease

#### 3.3.1. Age

We found that older patients were more likely to have CKD (67.22 ± 14.53 vs. 70.47 ± 10.94 years, *p* = 0.001). In addition, a higher CKD stage was observed at older age (CKD 1st stage 52.33 ± 31.53 years vs. CKD 4th stage 75.69 ± 13.67 years, 95% CI (0.48; 46.24), *p* = 0.042). CKD stage did not differ between the genders.

#### 3.3.2. Duration of Treatment

The duration of hospitalization depends on the stage of CKD (*p* = 0.023). Patients with the stage 5 CKD were hospitalized 1.2 times longer than patients with stage 3 CKD (26.44 ± 28.16 vs. 16.32 ± 14.12 days, 95% CI (0.40; 19.83), *p* = 0.036) and 2.04 times longer than patients with unspecified CKD stage (26.44 ± 28.16 vs. 12.95 ± 21.26 days, 95% CI (1.13; 25.78), *p* = 0.022).

#### 3.3.3. Outcomes

Patient outcomes depend on the presence of CKD (*p* < 0.001) and the stage of CKD (*p* < 0.001). Non-hospitalized emergency patients were more likely to have unspecified CKD stage in the medical records (*p* < 0.001). Patients with stage 3 CKD were more likely to be discharged home than to die in the hospital (*p* = 0.037).

### 3.4. Acute Kidney Injury

#### 3.4.1. Age

**Figure 1:**
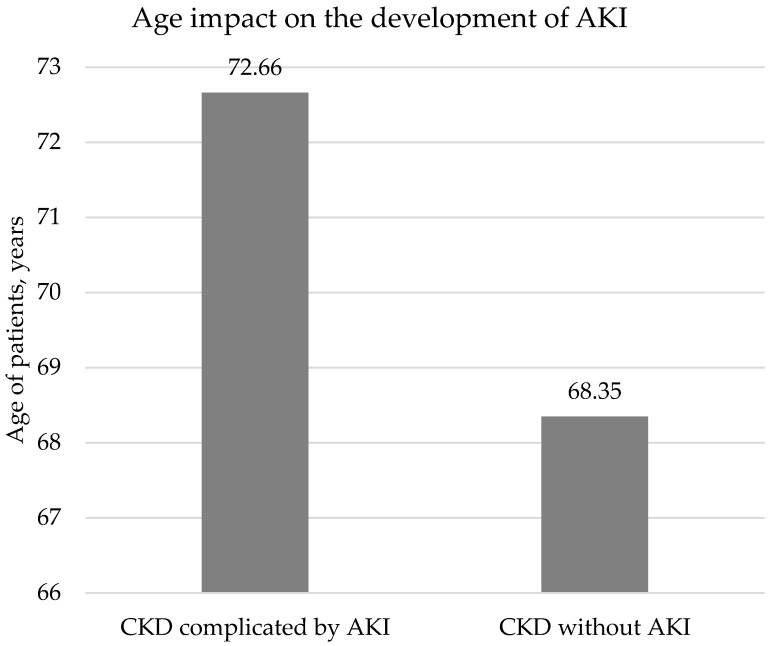
Age’s impact on the development of AKI.

We found that the older the patient, the more often CKD was complicated by AKI during COVID-19 infection (average age of patients with AKI 72.66 ± 11.98 years vs. patients without AKI 68.35 ± 14.86 years, *p* = 0.014) (Figure 1).

#### 3.4.2. Duration of Treatment

**Figure 2:**
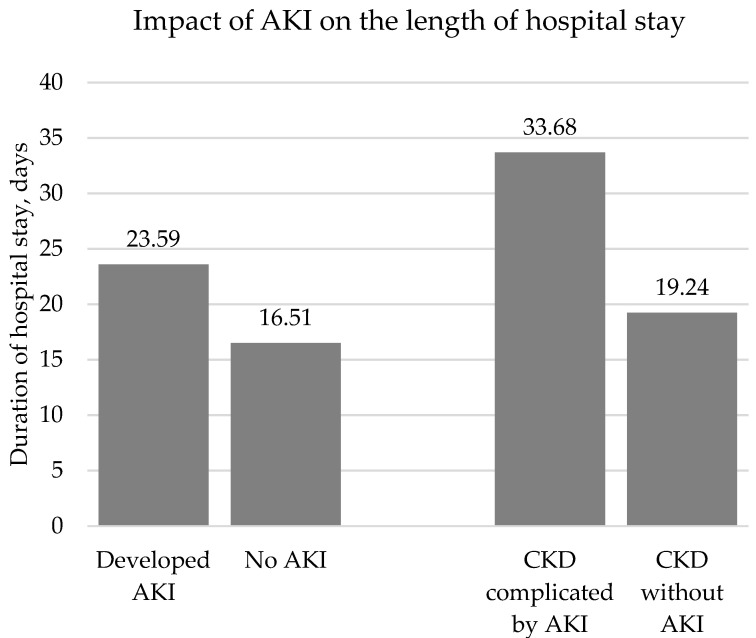
Impact of AKI on the length of hospital stay.

Patients who developed AKI during COVID-19 infection had a longer hospital stay (23.59 ± 25.53 days) than patients who did not develop AKI (16.51 ± 18.27 days, *p* < 0.001). Patients with CKD complicated by AKI had a longer hospital stay than those without AKI (33.68 ± 29.24 vs. 19.24 ± 32.84 days, *p* < 0.001) (Figure 2).

#### 3.4.3. Outcomes

Patients with CKD complicated by AKI had a statistically significant 3.66-fold higher incidence of death than patients without complications (hospitalization ended in death 3.66 times more (95% CI 1.96; 6.84, *p* < 0.001)) (Table 3).

Patients with AKI were 7.81 times (95% CI 5.18; 11.78, *p* < 0.001) more likely to die than patients with CKD alone (Table 3).

Patients with AKI were 7.79 times (95% CI 2.87; 21.19, *p* < 0.001) more likely to die than patients with neither CKD nor AKI (Table 3).

### 3.5. Hemodialysis

#### 3.5.1. Age

There were no significant differences between the different age groups with regard to HD treatment.

#### 3.5.2. Duration of Treatment

The length of hospital stay was longer in patients treated with HD than in patients not treated with HD (30.35 ± 29.05 vs. 17.10 ± 19.27 days, *p* < 0.001). The length of hospital stay was longer in patients with AKI treated with HD than in patients with CKD treated with HD (32.48 ± 28.20 vs. 22.00 ± 24.94 days, *p* = 0.022) but shorter in patients with CKD complicated by AKI (32.48 ± 28.20 vs. 50.60 ± 33.53 days, *p* = 0.010). The duration of treatment in CKD patients treated with HD was shorter than in patients with CKD complicated by AKI (22.00 ± 24.94 vs. 50.60 ± 33.53 days, *p* < 0.001) (Table 4).

#### 3.5.3. Outcomes

There was no overall higher rate of deaths among patients treated with HD (OR = 1.07, 95% CI (0.74; 1.54)), although patients with AKI treated with HD were 3.89 times more likely to die than those with CKD complicated by AKI (OR = 3.89, 95% CI (1.43; 10.57)). Patients with CKD were 1.54 times (95% CI (1.04; 2.03), *p* < 0.001) more likely to have HD than patients with AKI. Patients with CKD complicated by AKI were 2.53 times (95% CI (1.39; 4.62), *p* < 0.001) more likely to have HD than patients with AKI alone. We did not observe any gender differences in the use of HD.

## 4. Discussion

Renal impairment is one of the most common consequences of SARS-CoV-2 infection [13]. A meta-analysis of nearly 90,000 hospitalized patients with COVID-19 infection worldwide showed a prevalence of AKI ranging from 10.6% to 12.3% [3,13,14]. The highest incidence of AKI was found in transplanted and ICU patients, 38.9% (95% CI 27.3–51.9%) and 39.0% (95% CI 23.2–57.6%), respectively [14]. In our study, 57.6% of the included patients were diagnosed with AKI. The overall prevalence of AKI among hospitalized patients without COVID-19 is 8% [15].

### 4.1. Pathogenesis

SARS-CoV-2 is a cytopathic virus that enters the cell via the angiotensin-converting enzyme 2 (ACE2) membrane protein [16]. ACE2 is abundantly expressed in proximal renal tubule epithelial cells and podocytes, making these cells a frequent target for the virus. The transmembrane serine proteases (TMPRSS) of these cells are co-receptors and activate the spike protein on the surface of the SARS-CoV-2 virus and enable the virus to enter the cells [17]. Another study has shown that the SARS-CoV-2 virus enters target cells via the cell surface receptor CD147, which is also abundantly expressed in kidney cells [18,19]. The main pathological changes in the kidney cells are the degeneration of vacuoles, swelling of cells, the accumulation of the SARS-CoV-2 virus inclusion bodies in renal tubular epithelial cells, and the delamination of these cells [20,21]. In addition, the virus can cause tubule damage due to the membrane attack complex (MAC) and the infiltration of the tubule interstitium by CD68+ macrophages [22]. In a study by Wengqing Yin et al., in renal biopsies from patients with COVID-19, the most common finding was glomerular capillary disruption accompanied by hyperplasia of glomerular epithelial cells and abundant eosinophilic aggregates of intracytoplasmic protein [23]. This suggests that the SARS-CoV-2 virus directly infects and damages nephrons. Viral replication in target organs, including the kidney, leads to a systemic inflammatory response, viral sepsis, and multiple cellular lesions. SARS-CoV-2 can trigger a cytokine storm in which inflammatory factors such as IL-6, IL-1 β, TNF-α, and granulocyte colony-stimulating factor induce ischemia, hypoxia, fibrosis, rhabdomyolysis, intrarenal inflammation, increased vascular permeability, and renal damage [24,25,26]. In addition, hypoxemia, dehydration, inappropriate use of nonsteroidal anti-inflammatory drugs (NSAIDs), antibiotics, antivirals, and other potentially nephrotoxic drugs play a role in the pathogenesis of renal injury in COVID-19 patients [17,27]. In addition, co-morbidities such as diabetes mellitus and arterial hypertension may contribute to the development and severity of AKI [28]. Patients with CKD are at increased risk of severe viral infection due to a persistent proinflammatory state caused by defects in innate and adaptive immunity [6,29]. In a meta-analysis by Jagmeet Singh et al., CKD patients were almost twice as likely to develop severe COVID-19 infection (OR 1.97, 95% CI (1.61; 2.42)) [13].

### 4.2. Laboratory Findings

In a study by Li et al., 193 COVID-19 patients had elevated urea and creatinine concentration on the first day of hospitalization, in 14% and 10% patients, respectively [30]. Cheng et al. reported that patients with elevated creatinine and urea concentrations on the first day of hospitalization had a 2.10 (95% CI 1.36; 3.26) and 3.97 (95% CI 2.57; 6.14) times higher mortality, respectively. In addition, these patients were more frequently admitted to the ICU, required artificial ventilation, and deteriorated more often than patients with normal creatinine concentration [6]. Interestingly, in our study, patients who died had a lower creatinine concentration on the first day after admission to the regional hospital (185.00 µmol/L) than those who were discharged home (311.17 µmol/L) (95% CI (36.65; 215.70), *p* < 0.001). In addition, patients with higher creatinine (Spearman’s correlation coefficient = −0.304, *p* < 0.001) and urea (Spearman’s correlation coefficient = −0.247, *p* < 0.001) concentrations on the first day had a statistically significantly shorter hospital stay. This could be explained be the different creatinine concentrations on the first day in patients with chronic kidney disease and acute kidney injury (365.72 ± 311.93 vs. 137.58 ± 93.75 µmol/L, 95% CI (−303.37; −152.92), *p* < 0.001).

### 4.3. Age

In meta-analysis by Lirong Lin et al., age over 60 years in COVID-19 patients was an independent risk factor for AKI (OR 3.53, 95% CI (2.92; −4.25)) [3]. In our study, the mean age of the patients was 68.75 ± 14.66 years. Older age is associated with a weakened immune system and aging of tissues, which lead to increased susceptibility and sensitivity to the virus [31,32]. In our study, there was no statistically significant difference in age between patients with or without AKI (*p* = 0.502), but we found that CKD was more frequently complicated by AKI in older patients during COVID-19 infection (72.66 ± 11.98 vs. 68.35 ± 14.86 years, *p* = 0.014).

### 4.4. Outcomes

The literature shows that the mortality rate of patients not infected with SARS-CoV-2 in the case of AKI ranges from 1.0% to 14.4% in non-ICUs and up to 21.8% in ICUs [33]. In the meta-analysis of Lirong Lin et al., the mortality rate of hospitalized COVID-19 patients with AKI was 22.1%, which was 11.05 times higher than that of COVID-19 patients without AKI [3]. AKI was associated with a 5.3-fold increased risk of mortality in COVID-19 patients according to the study by Zhen Li et al. [30]. As reported by Xiaopeng Yang et al.’s study, 42% of patients who died during hospitalization were also diagnosed with AKI [14]. The results of our study are in line with these findings: the mortality rate in patients with AKI reached 34.94% and was 7.79 (95% CI 2.87; 21.19, *p* < 0.001) times higher than in patients without AKI.

In a meta-analysis of 15,000 COVID-19 patients by Jagmeet Singh et al., the prevalence of CKD was 9.7% [13]. In our study, 42.9% of patients had CKD. In another meta-analysis, patients with CKD had a 10.26-fold (95% CI (6.78; 15.53)) higher mortality rate than patients without CKD [34]. In another study involving of more than 4000 COVID-19 patients with kidney disease, 50% of those with CKD and on dialysis died within 28 days of admission to the ICU compared to 35% of patients without kidney disease [35]. In our study, the highest in hospital mortality rate was among patients with AKI alone (30.9%) and was 7.81 times (95% CI (5.18; 11.78), *p* < 0.001) higher than in patients with CKD alone. Patients with CKD complicated by AKI had a mortality rate that was 3.66 (95% CI (1.96; 6.84), *p* < 0.001) times higher than that of patients with CKD alone.

### 4.5. Gender

According to a meta-analysis, as in our study, the incidence of AKI did not differ between genders in COVID-19 patients (OR 1.36, 95% CI (0.84; 2.20)).

Some studies reported the odds of death were 11.05 times (95% CI (9.13; 13.36) higher among men with AKI [3]. Another study observed that the number of SARS-CoV-2 virus particles in the blood of male patients decreases much more slowly than in females, which may lead to a higher incidence of more severe symptoms and complications in men [36]. In addition, the higher prevalence of smoking and alcohol consumption, as well as biological differences in the immune system, such as the expression of androgen response elements (AREs) of the TMPRSS2 gene, which facilitates the entry of the virus into the epithelial cells, leads to difference in susceptibility of the two sexes to SARS-CoV-2 virus [37]. However, in our study, patient outcome and the incidence of AKI were not statistically significantly related to gender.

### 4.6. Hemodialysis

According to various studies, 1.5–9.0% (4.8% of non-transplanted and 15.6% of transplanted patients) [3,14] of COVID-19 patients require renal replacement therapy in therapeutic wards, while 5.6–23.0% require renal replacement therapy in ICUs [5,26,38,39,40]. In our study, 26.3% of patients underwent hemodialysis. Their duration of hospitalization was almost twice as long as patients without HD treatment (30.35 ± 29.05 vs. 17.10 ± 19.27 days, *p* < 0.001). In our study, HD was 1.54 times (CI 95% (1.04; 2.03), *p* < 0.001) more frequent in patients with CKD than those with AKI. HD was 2.53 times (CI 95% 1.39, 4.62, *p* < 0.001) more frequent in patients with CKD complicated by AKI than in those with AKI alone.

Renal replacement therapy is needed for both renal-related (e.g., treatment of acute renal failure with hemodynamic instability) and non-renal-related (e.g., difficult-to-control ARDS, hypernatremia, volume overload, diuretic resistance) complications [41]. Moreover, studies have shown that renal replacement therapy can non-selectively remove inflammatory mediators, regulate immune and hemodynamic stability, reduce excess fluid in the lungs, and achieve acid–base balance [42,43,44]. Therefore, the timely introduction of renal replacement therapy can improve the outcome of COVID-19 patients and reduce hospital costs.

## 5. Conclusions

Our study highlights the significant impact of kidney damage during COVID-19 infection on patients’ outcomes and hospitalization times. This study demonstrates the importance of measuring creatinine concentration on the first day of hospitalization as it helps to predict the length of hospitalization and higher probability of mortality. This study proved that older age is a risk factor for AKI as a complication of CKD in COVID-19 patients and a risk factor for longer hospitalization. Patients with AKI and those with CKD complicated by AKI are at the higher risk of longer hospitalization and death than patients with uncomplicated CKD or neither CKD nor AKI. Hemodialysis patients with AKI had a higher rate of hospitalization and death than patients with CKD complicated by AKI. We did not observe any differences between patients of different genders.

## Figures and Tables

**Table 1:** Characteristics of patients.

Characteristics		Values
Age, years *, N = 601		68.75 ± 14.66
Gender, N (%)	Male	351 (58.40)
	Female	250 (41.60)
Type of case, N (%)	Emergency patients—hospitalized	471 (78.37)
	Emergency patients—not hospitalized	64 (10.65)
	Inpatient patients	66 (10.98)
Duration of treatment *, days		20.59 ± 22.99
Outcomes, N (%)	Death	259 (43.09)
	Discharged from inpatient care home	229 (38.10)
	Went home from the emergency room	52 (8.65)
	Transferred to another hospital for an active inpatient treatment	42 (6.99)
	Transferred to the supportive care hospital	19 (3.16)
Patients with CKD, N= 258, N (%)	Stage 1	3 (1.11)
	Stage 2	19 (7.09)
	Stage 3	84 (31.34)
	Stage 4	36 (13.43)
	Stage 5	101 (43.69)
	Not specified	15 (5.59)
Patients with AKI, N = 346, N (%)	With tubular necrosis	4 (1.16)
	With acute cortical necrosis	1 (0.29)
	Not specified	341 (98.55)
Patients with CKD complicated by AKI, N (%)		56 (16.18)
Patients with unspecified kidney failure, N (%)		21 (3.49)
Patients undergoing hemodialysis, N (%)		158 (26.30)
Patients after kidney transplantation, N (%)		8 (1.33)

CKD—chronic kidney disease, AKI—acute kidney injury. * Average.

**Table 2:** Correlation between laboratory findings, age, and duration of treatment.

Value		Age	Duration of Treatment
Creatinine concentration	Spearman’s correlation coefficient	0.133	−0.304
	*p*-value	0.028	**<0.001**
Urea concentration	Spearman’s correlation coefficient	0.326	−0.247
	*p*-value	<0.001	**<0.001**
Potassium concentration	Spearman’s correlation coefficient	0.046	−0.057
	*p*-value	0.267	0.172
CRP concentration	Spearman’s correlation coefficient	0.005	−0.025
	*p*-value	0.899	0.548

**Table 3:** Impact of AKI and CKD on patient outcomes.

	Outcome		OR	95% CI
	Death	Survival		
CKD complicated by AKI	25	31	3.66	1.96; 6.84
CKD without AKI	42	186		
Only AKI	186	106	7.81	5.18; 11.78
Only CKD	42	187		
Only AKI	186	105	7.79	2.87; 21.19
No CKD or AKI	5	22		

**Table 4:** Difference in duration of treatment between HD-treated and non-HD-treated patients.

	Patients Treated with HD		*p*-Value
	HD-treated (N = 158, 26.30%)	Non-HD-treated (N = 443, 73.71%)	
Duration of treatment *, days	30.35 ± 29.05	17.10 ± 19.27	**<0.001**
	AKI patients treated with HD (N = 63, 10.48%)	CKD patients treated with HD (N = 67, 11.15%)	
	32.48 ± 28.20	22.00 ± 24.94	**0.022**
	AKI patients treated with HD (N = 63, 10.48%)	CKD patients complicated by AKI treated with HD (N = 23, 3.83%)	
	32.48 ± 28.20	50.60 ± 33.53	**0.010**
	CKD patients treated with HD (N = 67, 11.15%)	CKD patients complicated by AKI treated with HD (N = 23, 3.83%)	
	22.00 ± 24.94	50.60 ± 33.53	**<0.001**

* Average.

## Data Availability

The data presented in this study are available on request from the corresponding author if a proper procedure would be carried out. The data are not publicly available because it might compromise the privacy of the research participants.
